# Supplementary material for: Comprehensively Testing the Function of Missense Variation in the STK11 Tumour Suppressor
Source: bioRxiv. 2025 Jul 18:2025.07.14.664734. Preprint. [Version 1] doi: 10.1101/2025.07.14.664734 (PMC12338614; doi:10.1101/2025.07.14.664734)
Supplement: Supplement 2 [file NIHPP2025.07.14.664734v1-supplement-2.pdf]

bioRxiv preprint doi: https://doi.org/10.1101/2025.07.14.664734; this version posted July 18, 2025. The copyright holder for this preprint (which was not certified by peer review) is the author/funder, who has granted bioRxiv a license to display the preprint in perpetuity. It is made available under aCC-BY 4.0 International license.

Supplementary Figures

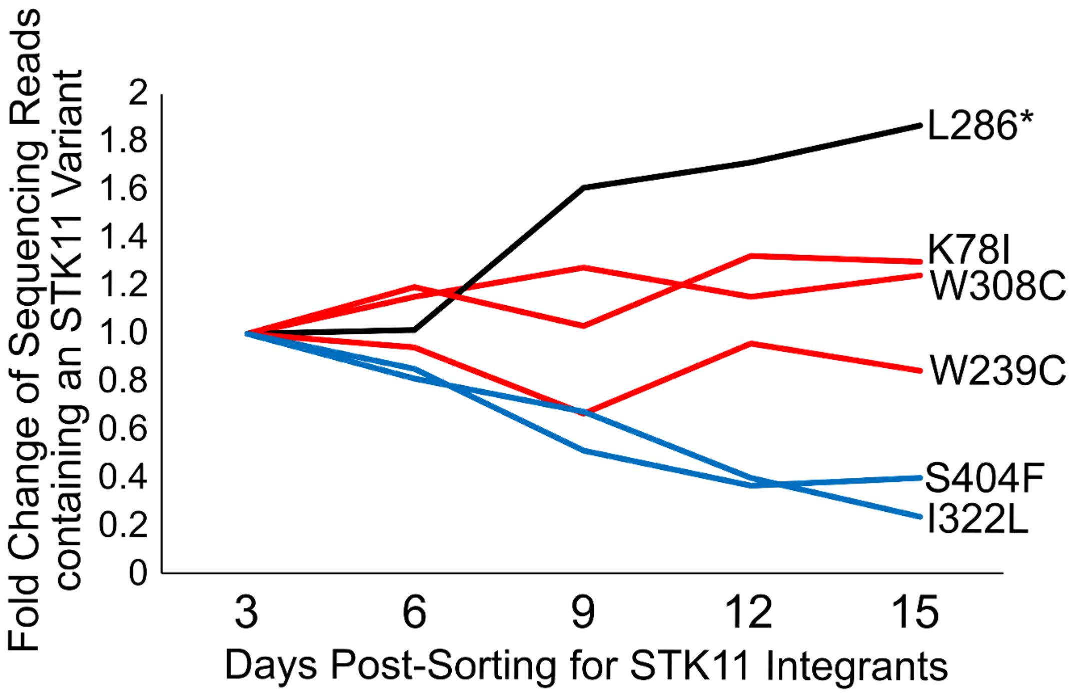

Supplementary Figure 1 Small-Scale Validation of the HeLa Proliferation Assay. Equimolar quantities of Bxb1 plasmids bearing *STK11* variants (K78I, W239C, L286*, W308C, I322L, and S404F) were transfected into HeLa cells for stable integration. Successful *STK11* integrants were isolated by FACS then grown in culture over two-week time with a portion of the cells harvested every three days for sequencing. As a proxy for proliferation rate, the change in frequency of each *STK11* variant in the pool was quantified by NGS at multiple time-points.

bioRxiv preprint doi: https://doi.org/10.1101/2025.07.14.664734; this version posted July 18, 2025. The copyright holder for this preprint (which was not certified by peer review) is the author/funder, who has granted bioRxiv a license to display the preprint in perpetuity. It is made available under aCC-BY 4.0 International license.

Missense variants expected to impact protein function are shown in red, nonsense variants in black, and tolerated missense in blue.

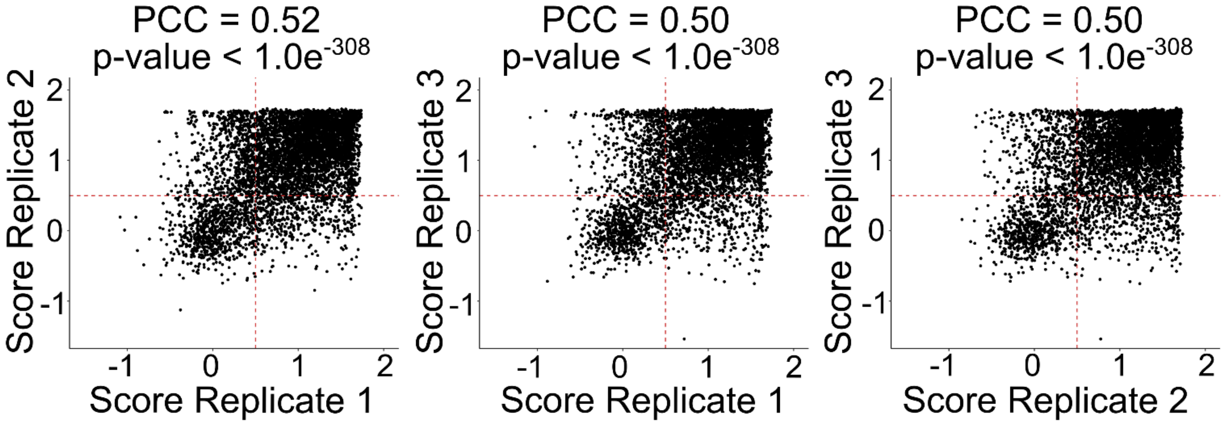

Supplementary Figure 2 Correlation of Functional Scores Across Biological Replicates. Missense variant functional scores from each of the three biological replicates (i.e. independent transfections) were plotted in pairwise combinations. Intermediate functional scores of 0.5 are indicated by horizontal and vertical red dashed lines. Pearson correlation was calculated, and the correlation coefficient and p-value are reported above the plots.

bioRxiv preprint doi: https://doi.org/10.1101/2025.07.14.664734; this version posted July 18, 2025. The copyright holder for this preprint (which was not certified by peer review) is the author/funder, who has granted bioRxiv a license to display the preprint in perpetuity. It is made available under aCC-BY 4.0 International license.

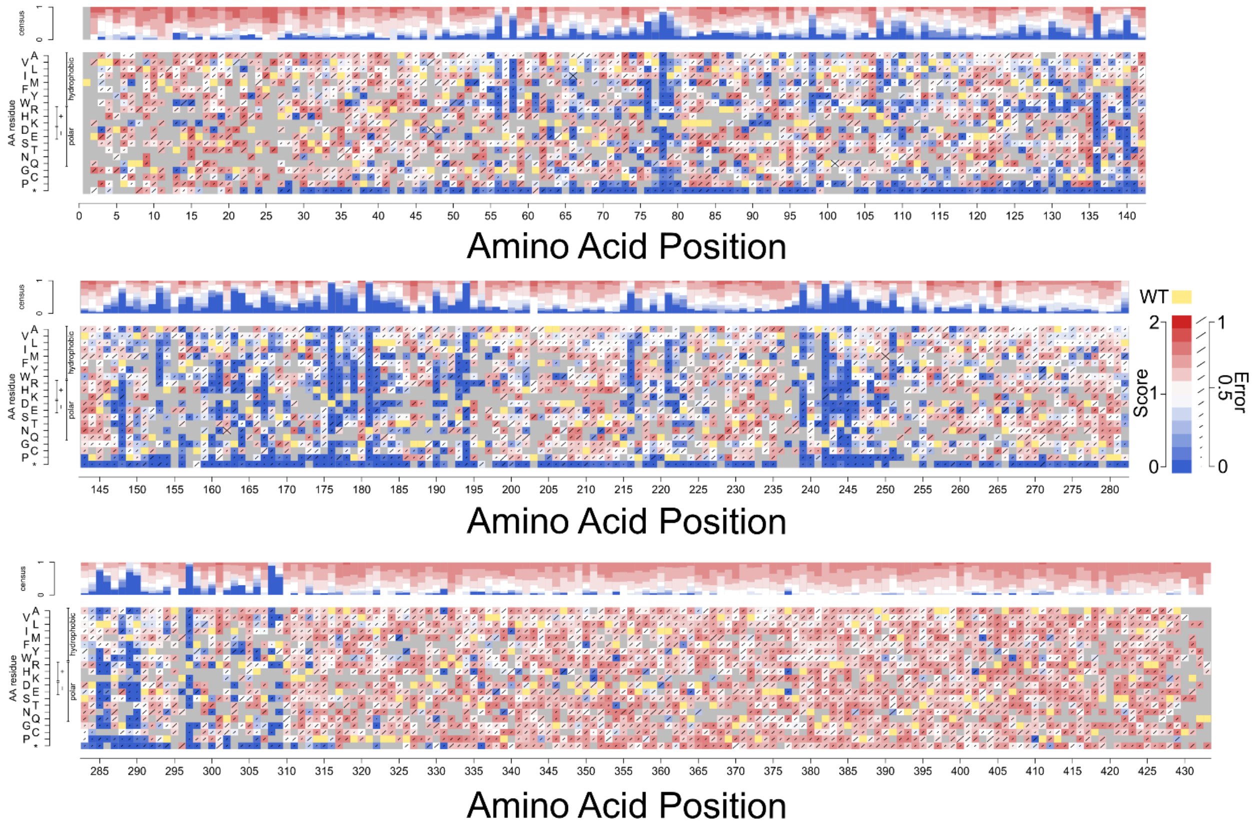

Supplementary Figure 3 Full-Length STK11 Variant Effect Map. Functional scores for all well-measured variants are shown as a heatmap with all possible amino acid substitutions on the y-axis, and position along STK11 on the x-axis. Yellow indicates the wild-type residue, gray for variants that did not pass filtering, blue for damaging scores near 0, white for tolerated scores near 1, red for scores above 1. Error rates (calculated as described in the methods) are shown as diagonal lines within each cell, with larger lines for higher error rates.

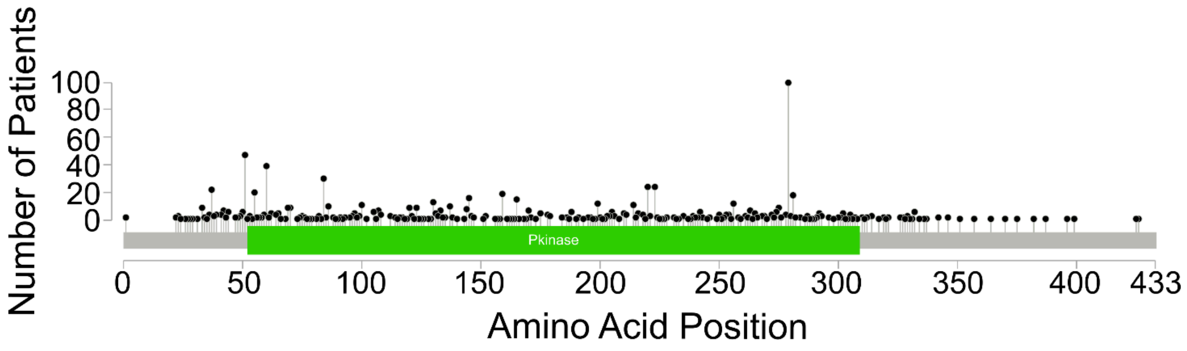

Supplementary Figure 4 STK11 Truncating Variants on cBioPortal. The curated set of non-redundant studies was selected on cBioPortal (As of May 2025) with a gene-specific query

bioRxiv preprint doi: https://doi.org/10.1101/2025.07.14.664734; this version posted July 18, 2025. The copyright holder for this preprint (which was not certified by peer review) is the author/funder, who has granted bioRxiv a license to display the preprint in perpetuity. It is made available under aCC-BY 4.0 International license.

for *STK11*. All unique *STK11* truncating variants were depicted as black dots on the lollipop plot, with the x-axis showing position along STK11, and the y-axis showing the number of patients with each variant.

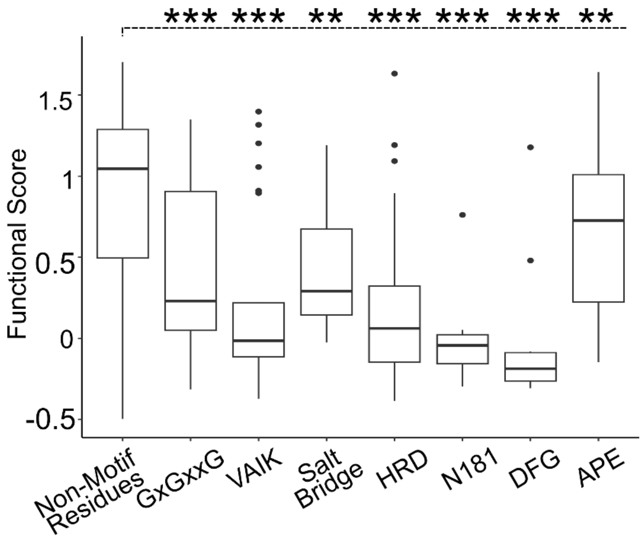

Supplementary Figure 5 Functional Scores at Conserved Motif Positions. Functional score for each variant at conserved kinase motif positions that matched the consensus sequence are shown. Boxes show the interquartile range between the 25th to 75th quartiles, with a solid horizontal line for the 50th percentile. Vertical lines extending from each box represent variants with scores within 1.5 times the interquartile range, and circular dots for variants exceeding that threshold.

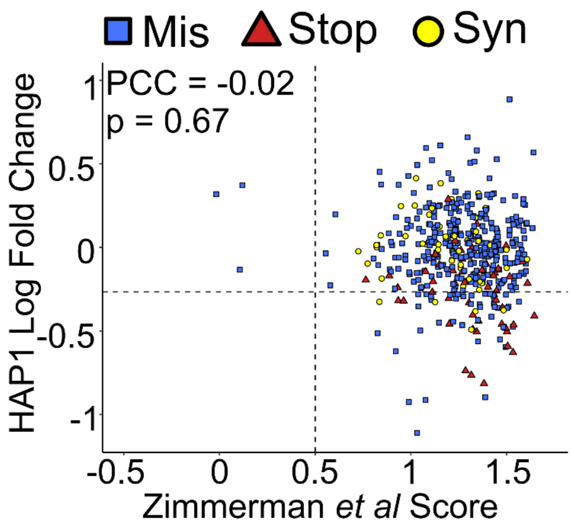

Supplementary Figure 6 *STK11* Exon 9 Essentiality Assay in HAP1 Cells. Functional scores

bioRxiv preprint doi: https://doi.org/10.1101/2025.07.14.664734; this version posted July 18, 2025. The copyright holder for this preprint (which was not certified by peer review) is the author/funder, who has granted bioRxiv a license to display the preprint in perpetuity. It is made available under aCC-BY 4.0 International license.

in exon 9 were matched to results from a *STK11* essentiality assay in HAP1 cells. Loss of *STK11* function in HAP1 cells reduces their ability to proliferate and survive, leading to drop-out in the pooled population. Missense variants are shown as blue squares, nonsense variants as red triangles, and synonymous variants as yellow circles.

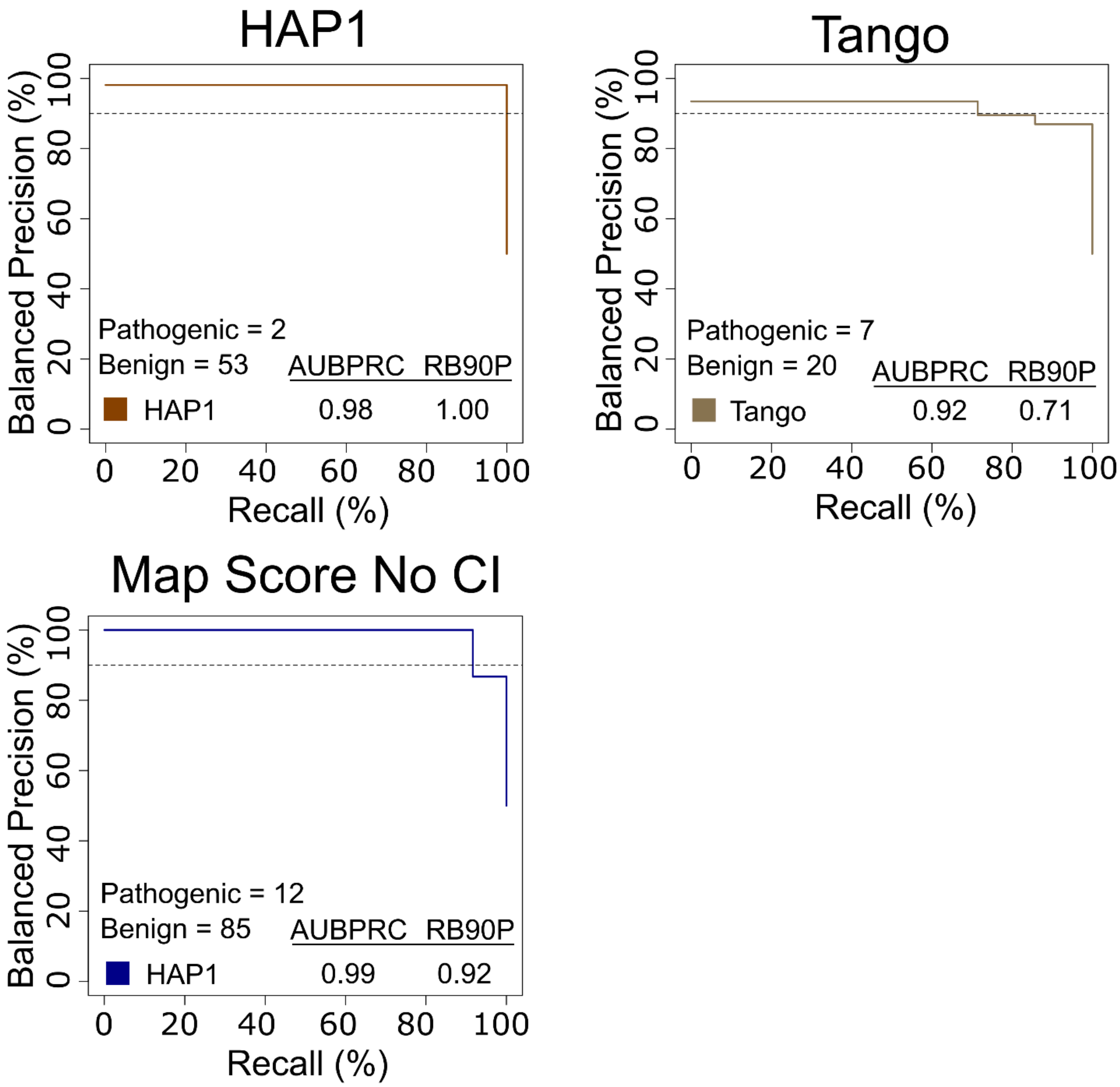

Supplementary Figure 7 Precision-Recall Curves for Alternative Assays. Precision-recall curves were generated for *STK11* functional scores from the HAP1 essentiality assay, the Tango xenograft, and all HeLa map scores without the confidence interval filter. Using a known set of clinically annotated pathogenic or benign *STK11* variants from Ambry Genetics, we evaluated balanced precision—defined at each score threshold by the fraction of variants that are pathogenic given a balanced (50% prior probability of pathogenicity) test set—versus recall

bioRxiv preprint doi: https://doi.org/10.1101/2025.07.14.664734; this version posted July 18, 2025. The copyright holder for this preprint (which was not certified by peer review) is the author/funder, who has granted bioRxiv a license to display the preprint in perpetuity. It is made available under aCC-BY 4.0 International license.

(fraction of pathogenic variants captured at this threshold). The horizontal dashed line indicates R90BP with the numerical AUPRC and R90BP listed in the bottom-left hand legend.

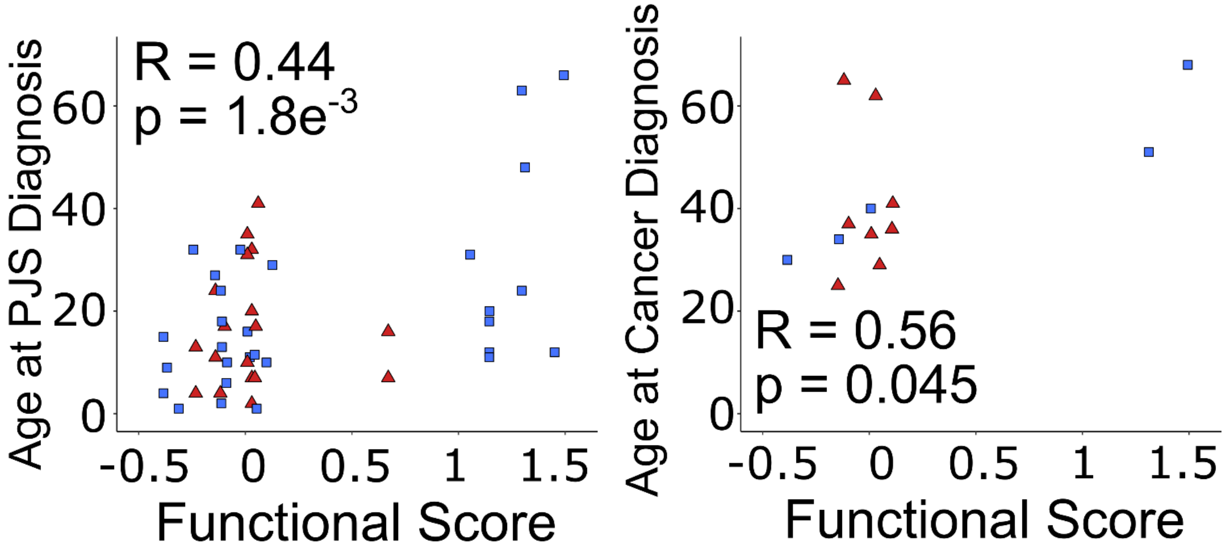

Supplementary Figure 8 Detrimental Functional Scores Are Associated with Earlier Diagnosis of PJS and Onset of Cancer. Age at initial PJS diagnosis and age at PJS-associated cancer were collected from patient charts at the Zane Cohen Centre as well as from a literature review of available PJS case studies. Red triangles indicate nonsense variants, blue squares represent missense variants. The age at first diagnosis was determined by the earliest manifestation of recorded symptoms (such as hyper-pigmentation, observation of intestinal polyps, intussusception, etc.). Pearson correlation between age at PJS diagnosis or age at PJS-associated cancer and functional score is provided.

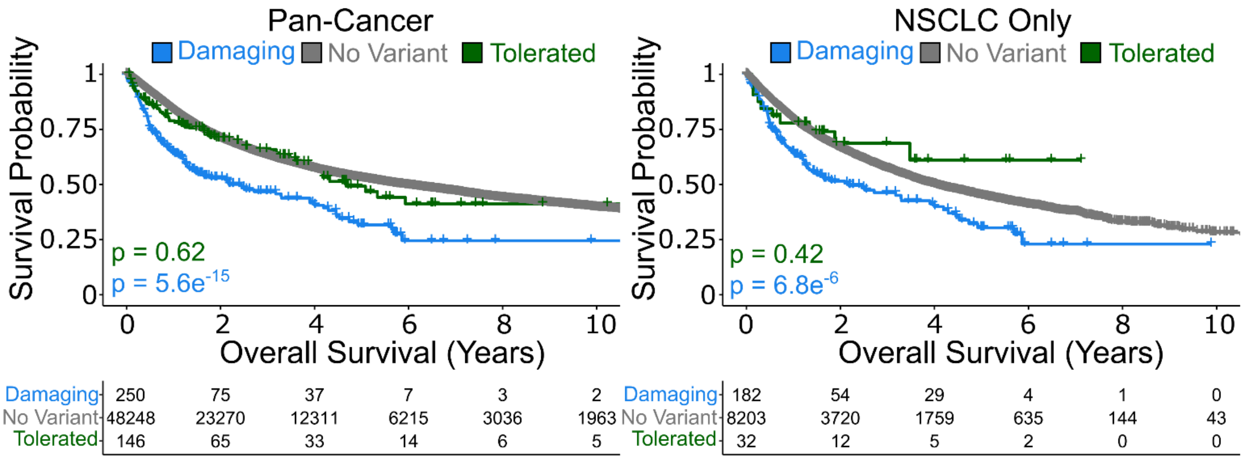

Supplementary Figure 9 Stratifying Somatic Cancers by Functional Score in cBioPortal.

bioRxiv preprint doi: https://doi.org/10.1101/2025.07.14.664734; this version posted July 18, 2025. The copyright holder for this preprint (which was not certified by peer review) is the author/funder, who has granted bioRxiv a license to display the preprint in perpetuity. It is made available under aCC-BY 4.0 International license.

Metrics relating to overall survival for individuals with cancer were extracted from a non-redundant set of clinical studies on cBioPortal (as of March 2025). Datasets on cBioPortal directly provided metrics of overall survival, here displayed in years. Patients were stratified into those without an *STK11* variant (gray), a tolerated *STK11* variant (green), or a damaging *STK11* variant (blue) according to our map score. Kaplan-Meier survival curves visualized the probability of survival over time in years, each 'drop' representing a death end-point, and each 'tick' a currently living individual. Log-rank tests were performed to compare outcomes for individuals with a damaging or tolerated *STK11* variant against those with wild-type *STK11*.

Supplementary Tables

	Fraction of All Possible Missense Variants	Fraction of SNV-Reachable Missense Variants
**Replicate 1**	74.7% (6,129)	80.2% (2,061)
**Replicate 2**	75.2% (6,170)	83.0% (2,132)
**Replicate 3**	73.4% (6,021)	79.6% (2,043)
**Final Combined Score**	73.4% (6,026)	79.8% (2,049)

Supplementary Table 1. Coverage of *STK11* Missense Variants in Each Biological Replicate. Each biological replicate consists of three independent transfections (one per mutagenesis region of *STK11*) of the HeLa proliferation assay. The fraction of all possible *STK11* missense variants (8,208 possible variants), and the fraction of single-nucleotide accessible variants (2,568 possible variants) is shown along with the number of variants in brackets.

bioRxiv preprint doi: https://doi.org/10.1101/2025.07.14.664734; this version posted July 18, 2025. The copyright holder for this preprint (which was not certified by peer review) is the author/funder, who has granted bioRxiv a license to display the preprint in perpetuity. It is made available under aCC-BY 4.0 International license.

**Wild-Type**<br>***STK11*-201**<br>**ENST00000326873.12**<br>**Q15831-1**<br>**NM_000455.5**	ATGGAGGTGGTGGACCCGCAGCAGCTGGGCATGTTCACGGAGG<br>GCGAGCTGATGTCGGTGGGTATGGACACGTTCATCCACCGCATC<br>GACTCCACCGAGGTCATCTACCAGCCGCGCCGCAAGCGGGCCA<br>AGCTCATCGGCAAGTACCTGATGGGGGACCTGCTGGGGGAAGG<br>CTCTTACGGCAAGGTGAAGGAGGTGCTGGACTCGGAGACGCTGT<br>GCAGGAGGGCCGTCAAGATCCTCAAGAAGAAGAAGTTGCGAAGG<br>ATCCCCAACGGGGAGGCCAACGTGAAGAAGGAAATTCAACTACT<br>GAGGAGGTTACGGCACAAAAATGTCATCCAGCTGGTGGATGTGTT<br>ATACAACGAAGAGAAGCAGAAAATGTATATGGTGATGGAGTACTGC<br>GTGTGTGGCATGCAGGAAATGCTGGACAGCGTGCCGGAGAAGC<br>GTTTCCCAGTGTGCCAGGCCCACGGGTACTTCTGTCAGCTGATT<br>GACGGCCTGGAGTACCTGCATAGCCAGGGCATTGTGCACAAGGA<br>CATCAAGCCGGGGAACCTGCTGCTCACCACCGGTGGCACCCTCA<br>AAATCTCCGACCTGGGCGTGGCCGAGGCACTGCACCCGTTCGC<br>GGCGGACGACACCTGCCGGACCAGCCAGGGCTCCCCGGCTTTC<br>CAGCCGCCCGAGATTGCCAACGGCCTGGACACCTTCTCCGGCTT<br>CAAGGTGGACATCTGGTCGGCTGGGGTCACCCTCTACAACATCA<br>CCACGGGTCTGTACCCCTTCGAAGGGGACAACATCTACAAGTTGT<br>TTGAGAACATCGGGAAGGGGAGCTACGCCATCCCGGGCGACTGT<br>GGCCCCCCGCTCTCTGACCTGCTGAAAGGGATGCTTGAGTACGA<br>ACCGGCCAAGAGGTTCTCCATCCGGCAGATCCGGCAGCACAGCT<br>GGTTCCGGAAGAAACATCCTCCGGCTGAAGCACCAGTGCCCATC<br>CCACCGAGCCCAGACACCAAGGACCGGTGGCGCAGCATGACTG<br>TGGTGCCGTACTTGGAGGACCTGCACGGCGCGGACGAGGACGA<br>GGACCTCTTCGACATCGAGGATGACATCATCTACACTCAGGACTT<br>CACGGTGCCCGGACAGGTCCCAGAAGAGGAGGCCAGTCACAAT<br>GGACAGCGCCGGGGCCTCCCCAAGGCCGTGTGTATGAACGGCA<br>CAGAGGCGGCGCAGCTGAGCACCAAATCCAGGGCGGAGGGCCG<br>GGCCCCCAACCCTGCCCGCAAGGCCTGCTCCGCCAGCAGCAAG<br>ATCCGCCGGCTGTCGGCCTGCAAGCAGCAGTGA
**Codon Optimized**	ATGGAAGTGGTGGATCCGCAGCAACTGGGAATGTTCACGGAAGG<br>AGAGCTGATGTCGGTGGGTATGGACACGTTCATCCACCGCATCGA<br>CTCCACAGAGGTCATCTACCAGCCTAGACGCAAGAGGGCCAAGC<br>TCATAGGTAAGTATCTGATGGGGGATCTGCTGGGAGAAGGCTCTT<br>ACGGCAAAGTGAAGGAGGTTCTGGACTCGGAGACTCTTTGCAGG<br>AGAGCCGTCAAGATCCTCAAGAAGAAGAAGTTGCGAAGGATCCC<br>CAACGGGGAAGCCAACGTGAAGAAGGAGATTCAACTACTGAGGA<br>GGTTACGGCACAAGAATGTCATCCAGCTGGTGGACGTGCTGTAC<br>AACGAGGAGAAGCAGAAGATGTACATGGTGATGGAGTACTGCGT<br>GTGTGGCATGCAGGAAATGCTGGACAGCGTGCCTGAGAAGCGTT<br>TCCCAGTGTGTCAGGCTCACGGGTACTTCTGTCAGCTGATTGACG<br>GCCTGGAGTACCTGCATAGCCAGGGCATTGTGCACAAGGACATC<br>AAACCGGGAAACCTGCTGCTCACCACAGGTGGAACTCTCAAAAT<br>CTCCGATCTGGGAGTGGCTGAAGCACTGCATCCTTTCGCTGCAG<br>ATGATACATGTCGGACCAGCCAAGGATCTCCTGCTTTCCAGCCTC<br>CAGAGATTGCCAATGGACTGGATACCTTCTCCGGCTTCAAGGTAG

bioRxiv preprint doi: https://doi.org/10.1101/2025.07.14.664734; this version posted July 18, 2025. The copyright holder for this preprint (which was not certified by peer review) is the author/funder, who has granted bioRxiv a license to display the preprint in perpetuity. It is made available under aCC-BY 4.0 International license.

	ACATCTGGTCAGCTGGGGTCACACTCTACAACATCACCACGGGTC<br>TGTACCCCTTCGAAGGGGACAACATCTACAAGCTGTTCGAGAACA<br>TCGGGAAGGGGAGCTATGCCATTCCAGGTGATTGTGGACCTCCG<br>CTCTCTGACCTGCTGAAAGGGATGCTTGAGTACGAACCTGCCAA<br>GAGGTTCTCCATCCGACAGATCAGACAGCACAGCTGGTTCCGGA<br>AGAAACATCCTCCTGCTGAAGCACCAGTGCCAATTCCACCGTCTC<br>CAGACACCAAAGACAGATGGAGGAGCATGACTGTGGTGCCTTAC<br>TTGGAGGATCTGCACGGAGCTGACGAGGATGAGGACCTCTTCGA<br>CATCGAGGACGACATCATCTACACTCAGGACTTCACGGTGCCTGG<br>ACAAGTCCCAGAAGAAGAGGCAAGTCACAATGGACAGCGCAGAG<br>GACTCCCCAAAGCTGTGTGTATGAACGGCACAGAGGCTGCACAG<br>CTGAGCACCAAATCCAGAGCGGAAGGTAGAGCTCCTAACCCTGC<br>CAGAAAGGCTTGCTCAGCCAGCAGCAAGATCAGAAGGCTGTCAG<br>CCTGCAAGCAGCAGTGA

Supplementary Table 2. Codon Optimized *STK11* cDNA Sequence. To facilitate mutagenesis of *STK11* (Transcript ENST00000326873.12, RefSeq NM_000455.5), we codon-optimized to ensure GC-content was consistently between 40-60% for each 33bp stretch across the gene.
